# Performance of PI-FAB and TARGET scoring systems in post focal therapy mpMRI surveillance

**DOI:** 10.21203/rs.3.rs-10581647/v1

**Published:** 2026-08-10

**Authors:** J. Bradley Mason, Jorge Arias, Bora Kalaycioglu, Aytekin Oto, Alejandro Calvillo Ramirez, Simon Han, Alex Weiss, Haidy Megahed, Arieh Shalhav, Ahmed Hamimi, Sang Mee Lee, Abhinav Sidana

**Affiliations:** University of Chicago; University of Chicago; University of Chicago; University of Chicago; University of Chicago; University of Chicago; University of Chicago; University of Chicago; University of Chicago; University of Chicago; University of Chicago; University of Chicago

**Keywords:** prostate cancer, ablation, PI-RADS, PI-FAB, TARGET

## Abstract

**Background:**

The Prostate Imaging after Focal Ablation (PI-FAB) and Transatlantic Recommendations for Prostate Gland Evaluation with Magnetic Resonance Imaging After Focal Therapy (TARGET) scoring systems have been recently developed to improve detection of post-ablation recurrence of clinically significant prostate cancer (csPCa). However, it is unknown how these two scoring systems compare in clinical practice.

**Methods:**

Patients who underwent focal therapy (FT) for prostate cancer (PCa) and had post-FT MRI and biopsy were identified from a prospective registry. Post-FT MRIs were reviewed by two radiologists blinded to biopsy results using the PI-FAB and TARGET systems with scores of 3 and ≥ 4, respectively considered positive for csPCa. csPCa recurrence was defined as ≥Gleason 3 + 4 on biopsy. Diagnostic performance metrics including sensitivity/specificity/positive predictive value (PPV)/negative predictive value (NPV)/accuracy were calculated. Area under the receiver operating characteristic curve (AUC) was generated using logistic regression and compared using DeLong’s tests. Inter-reader agreement was assessed with Cohen’s Kappa (κ).

**Results:**

90 patients were eligible. Radiologist #1 showed PI-FAB sensitivity/specificity/PPV/NPV/accuracy/AUC of 67%/82%/54%/89%/78%/74%, respectively; TARGET had values of 67%/79%/50%/88%/76%/73%, respectively. Radiologist #2 showed PI-FAB sensitivity/specificity/PPV/NPV/accuracy/AUC of 62%/68%/42%/83%/66%/65%, respectively; TARGET 62%/68%/42%/83%/66%/66%. Logistic regression showed no difference in AUC for PI-FAB and TARGET (0.69;95%CI:0.59–0.79 vs 0.71;95%CI:0.62–0.80, p = 0.696). Inter-reader agreement was fair for PI-FAB (k = 0.352, p = 0.02) and moderate for TARGET (k = 0.445, p < 0.01).

**Conclusion:**

PI-FAB and TARGET scoring systems perform similarly in diagnostic performance, though inter-reader agreement is limited. Clinical parameters may be needed to fully assess csPCa recurrence after focal therapy.

## Introduction

Focal therapy (FT) has become an attractive treatment option for patients with localized prostate cancer (PCa), providing oncologic control and improved functional outcomes compared to whole-gland treatments.^1,2^ Patients undergoing focal therapy (also known as partial ablation), including high intensity focused ultrasound (HIFU), TULSA-PRO (Transurethral Ultrasound Ablation of the prostate), cryotherapy, and irreversible electro-corporation (IRE) among others, showed no significant impact on urination 12 months, and 90% of potent men retained their potency.^3^ However, surveillance is critical to closely monitor patients undergoing focal therapy due to relatively high recurrence rates compared to radical treatment.^4^ Multiparametric MRI (mpMRI) has improved detection and assessment of clinically significant PCa (csPCa) over traditional transrectal ultrasound-guided biopsy in the treatment naive setting, with transrectal biopsies missing up to 30% of clinically significant PCa.^5–8^

While PIRADS (Prostate Imaging-Reporting and Data System, version 2) is currently used for assessment of index prostatic lesions, there is evidence that is should not be used in post-focal therapy surveillance imaging.^9,10^ PI-RADS is not optimized for post-ablative imaging, as artifacts from focal therapy (e.g. edema, inflammation or vascular changes) may be misattributed to post-treatment changes, potentially underdiagnosing csPCa recurrence.^11^ Indeed, PI-RADS states that the system should not be applied for the detection of tumor recurrence within ablated tissue after focal therapy, because the MRI findings of greatest concern are altered in this setting.^12^ There currently exists a scoring system to assess cancer recurrence in post radiation therapy or radical prostatectomy (Prostate Imaging for Recurrence Reporting),^13^ yet there is no consensus system for post focal therapy imaging. Detecting in-field recurrence post focal therapy significantly changes management, as these patients often need subsequent salvage treatment.^14^ Recently, the Prostate Imaging after Focal Ablation (PI-FAB) and Transatlantic Recommendations for Prostate Gland Evaluation with Magnetic Resonance Imaging After Focal Therapy (TARGET) scoring systems have been developed to address this need.

However, there are few studies directly comparing these post-FT scoring systems in clinical practice, leaving a knowledge gap for surveillance imaging. Current literature investigating PI-FAB and TARGET’s diagnostic performance shows no difference between the two scoring systems in detecting csPCa within the ablation zone, though a range of inter-reader variability exists.^16^ The most recent studies are also limited by small sample sizes and evaluate single-energy modalities only.^17–19^

To address these gaps, we performed a retrospective study to compare the diagnostic performances of PI-FAB and TARGET scoring systems in detecting csPCa, and to assess inter-reader agreement between two radiologists among patients undergoing FT with multiple energy modalities. Better understanding of these two scoring systems may help to improve post-ablation surveillance and identify patients who require salvage treatment.

## Methods

### Study Population

An IRB-approved prospective registry of patients who underwent FT for PCa at an academic center from 2020–2025 was retrospectively reviewed. Demographics, tumor characteristics, and follow up data were collected. Patients were included if they had both post-treatment mpMRI and surveillance biopsy performed within a 6-month window after ablation.

Study participants received partial gland FT via one of five energy modalities: high-intensity ultrasound (HIFU), cryoablation, irreversible electroporation (IRE), transurethral ultrasound ablation of the prostate (TULSA-PRO), or water vapor ablation. Postoperative follow-up visits were scheduled every 3 months during the first year and every 6 months thereafter. Prostate-specific antigen (PSA) levels were recorded at each visit. Multiparametric MRI was routinely performed between 6 and 12 months post-ablation per institutional protocols. At the 1-year follow-up, a transperineal or transrectal fusion prostate biopsy with targeted and systematic 12 cores was performed per protocol or at the surgeon’s discretion. For-cause biopsies were typically in the setting of rising PSA concentrations or suspicious post-ablation MRI findings. Patients were excluded from this study if no post-op MRI or biopsy was available, or if the post-op biopsy was performed more than 6-month after surveillance MRI (Fig. 1).

Post-treatment mpMRI images were independently interpreted by two experienced genitourinary radiologists at our institution, each blinded to biopsy results. Ablation zones were scored using the PI-FAB and TARGET systems with scores of 3 and ≥ 4, respectively, considered positive for csPCa per published data and paired to biopsy results.^14,15^ The remaining untreated prostate was scored with the PI-RADS scoring system, however as ablation zone recurrences and the performance of ablation zone scoring systems (i.e., PI-FAB and TARGET) were the focus of this study, MRIs were only considered positive if there were findings within the prior ablation zone. Biopsies were considered positive if csPCa (Gleason ≥ 3 + 4) was detected in the prior ablation zone (in-field); biopsy-proven recurrences outside of the ablation zone were not included in this analysis. Patients were excluded if they had whole-gland or subtotal ablation. A model was created that included the interpreter as a covariate to adjust for differences in radiologist experience and interpretation.

### Outcomes and Statistical Analysis

The primary outcome of our study was the diagnostic assessment of the PI-FAB and TARGET scoring systems. This was assessed by evaluating the sensitivity, specificity, positive predictive value (PPV), negative predictive value (NPV), accuracy of each scoring system, compared to post-ablation biopsy results as a gold standard. Accuracy is defined by PI-FAB/TARGET predicting a positive lesion when disease is truly present or predicting a negative lesion when disease is truly absent. In order to most-accurately match real-world clinical decision making, each post MRI reading was matched with one post op biopsy result using a 6-month window. Secondary outcomes included comparative analysis of PI-FAB and TARGET scoring systems, and inter-reader agreement between the reading radiologists. Because this study utilized a fixed cohort of all eligible patients from a prospective registry who met our strict inclusion criteria over a 5-year period, a formal *a priori* power analysis was not conducted. We acknowledge that our sample size may limit our ability to detect minor differences in diagnostic performance between the PI-FAB and TARGET systems.

For each scoring system, logistic regression models were fitted with the radiologist included as a fixed effect. From these models, the area under the receiver operating characteristic curve (AUC) was calculated, and AUCs for the two scoring systems were compared using Delong’s test. Diagnostic performance metrics—including sensitivity, specificity, accuracy, positive predictive value (PPV), negative predictive value (NPV) and area under the curve (AUC) were calculated separately for each radiologist and scoring system. We also conducted Chi-square tests between biopsy results and each MRI interpretation result to determine an association between binary results (positive or negative lesion). Inter-reader agreement was assessed for each scoring system using Cohen’s Kappa (k) statistics, and differences in agreement between scoring systems were evaluated using a nonparametric bootstrap approach with 2,000 resamples to account for the correlation between ratings. All statistical analyses were carried out using R software, version 4.3.2. Hypothesis testing was 2-sided, with a threshold of 0.05 set for statistical significance.

## Results

We identified 127 patients who underwent focal treatment during the study period. 90 patients were eligible for inclusion with available post-ablation MRI and biopsy (Fig. 1). Baseline characteristics are reported in Table 1. The median age was 69 (IQR 64–72) years, median PSA was 6.02 (4.66–8.12) ng/mL and 29 (32%) patients had initial Gleason grade group 3–5.

Of 90 patients, 57 (63.3%) underwent HIFU, 12 (13.3%) cryoablation, 12 (13.3%) water vapor ablation, 8 (8.9%) irreversible electroporation, and 1 (1%) had TULSA-PRO. Patients predominantly underwent primary treatment (12.2% underwent salvage ablation), with a median of one MRI lesion (IQR 1–2), median PI-RADS score of 4 (4–5). Most patients had GG1–2 disease (68%). 22 patients (24%) were positive for csPCa in the ablation zone confirmed by post-op biopsy (Table 1).

### Diagnostic Performance

Diagnostic performance by radiologist and scoring system are displayed in Table 2. Results from radiologist #1 show that PI-FAB had an sensitivity/specificity/PPV/NPV/accuracy/AUC of 67% / 82% / 54% / 89% / 78% / 74%, respectively. TARGET showed a sensitivity/specificity/PPV/NPV/accuracy/AUC of 67% / 79% / 50% / 88% / 76% / 73%, respectively. Results from radiologist #2 show that PI-FAB had a sensitivity/specificity/PPV/NPV/accuracy/AUC of 62% / 68% / 42% / 83% / 66% / 65%, respectively. For radiologist #2, TARGET sensitivity/specificity/PPV/NPV/accuracy/AUC of 62% / 68% / 42% / 83% / 66% / 66%. There was no statistically significant difference between AUC curves for the PI-FAB and TARGET (0.6916, 95% CI: 0.59–0.79 vs 0.7090, 95% CI: 0.62–0.80: p = 0.696) (Fig. 2). Scoring by radiologist is additionally illustrated in Fig. 3. A representative PI-FAB 3 and TARGET 5 lesion is illustrated in Fig. 4. On logistic regression, TARGET score showed an odds ratio (OR) of 5.45 (95% CI 2.60–11.79; p<.0001) for predicting csPCA recurrence, while PI-FAB had an OR of 5.53 (95% CI 2.63–12.08; p<.0001).

### Inter-reader Agreement

Inter-reader agreement was assessed with unweighted κ statistics. κ for PI-FAB and TARGET were 0.352 and 0.445, respectively, indicating fair agreement for PI-FAB and moderate agreement for TARGET.

## Discussion

In our study, we found high specificity and NPV for both scoring systems, and no difference between PI-FAB and TARGET scoring systems in detecting csPCa recurrences. There was moderate inter-reader agreement. We additionally show that positive scores are associated with high odds ratios of detecting csPCa on post-op biopsy.

Prior literature suggests similar concordance between the two scoring systems.^16–19^ A recent study assessing TARGET showed a high NPV and moderate inter-reader agreement, similar to our findings, though they report on a HIFU-only cohort with a smaller sample size.^16^ Gelikman et al also found that PI-FAB had a moderate inter-reader agreement and high sensitivity, though the scoring system was limited by low specificity and PPV.^17^ Pausch et al compared PI-FAB vs TARGET showing higher overall specificities (TARGET: 92%/89%; PI-FAB: 90%/90% for Reader 1/Reader 2) than our study, though NPV values (TARGET: 87%/81%; PI-FAB: 84%/80%) are similar to our findings. Sensitivity in our cohort was higher across both readers and scoring systems than those found by Pausch et al (TARGET: 55% in Radiologist 1, 35% in Radiologist 2; PI-FAB: 45% in Radiologist 1/30% in Radiologist 2).^18^ Consistent with our results, they report no significant difference between the scoring systems, though we show this in a larger sample size and across multiple energy modalities. Similarly, Esengur et al compared PI-FAB and TARGET after focal therapy in a cohort of 38 patients. Here, they found PI-FAB and TARGET exhibited similar performances in post-FT MRI and moderate agreement levels.^19^ Our study confirms the utility of these scoring systems among multiple treatment modalities (e.g., HIFU, cryoablation, IRE, etc.) and a larger sample size.

The lower sensitivities found in our study and others suggest these scoring systems do not optimally rule out csPCa, and MRI is susceptible to false negative results after focal therapy, highlighting the need for close surveillance protocols. Incorporation of clinical parameters such as PSA velocity or PSA density will be critical to further characterizing these findings. However, high NPV across both readers indicate that negative results with either scoring system correlates well with biopsy outcomes.

Although TARGET marginally outperformed PI-FAB on inter-reader agreement, overall agreement for both scales were only fair to moderate. While both radiologists have years of experience reading post-ablation imaging, the inherent novelty of these scoring systems likely contributed to these results. Prior to introduction of these scoring systems, a descriptive interpretation of the ablation zone based on early contrast enhancement was used to evaluate for recurrence. Inter-reader agreement is likely to improve in future studies as radiologists gain more experience in using these novel systems in post-FT MRI.

This study should be evaluated in the context of its limitations. We recruited only two radiologists from one academic center. However, to our knowledge, there are currently no multi-center studies evaluating PI-FAB and TARGET’s diagnostic performance and inter-reader agreement analyses are often limited to two radiologists.^18,19^ Additionally, in our study, each radiologist scored mpMRIs with both scoring systems in the same session, potentially introducing confirmation bias and leading to no difference between the two scoring systems. A washout time at staggered sessions could alleviate this concern. Larger, multi-reader, multicenter studies, ideally with blinded, independent reads at staggered sessions are needed to more accurately assess diagnostic performance and inter-reader agreement. Our study was also performed at a high-volume center for focal ablation, which may limit generalizability to other centers. However, as we have shown PI-FAB and TARGET scoring systems to be concordant, this may improve dissemination of either technique as focal ablation becomes more prevalent in the community. Our large population of patients who have undergone diverse modalities of focal ablation followed by MRI and biopsy is a major strength of the study compared to smaller cohorts in previous literature. Future work is needed to explore how PSA and other clinical parameters, in combination with PI-FAB and TARGET scores, change diagnostic accuracy of detecting csPCa.

These limitations notwithstanding, PI-FAB and TARGET show no difference in diagnostic performance metrics and positive scores are highly associated with cancer recurrence as calculated by OR. However, sensitivities, positive predictive values, and overall inter-reader agreement were moderate, highlighting the need for additional clinical parameters (e.g., PSA velocity, PSA density) to give further context in the surveillance setting. Our findings may help guide clinical decision making after ablation and improve outcomes for focal therapy patients and utilization of novel scoring systems after focal therapy. Future studies are needed to assess how these scoring systems change management after FT, and what impact combining these scores with other clinical parameters have on accurate detection of csPCa recurrences.

## Figures and Tables

**Figure 1:**
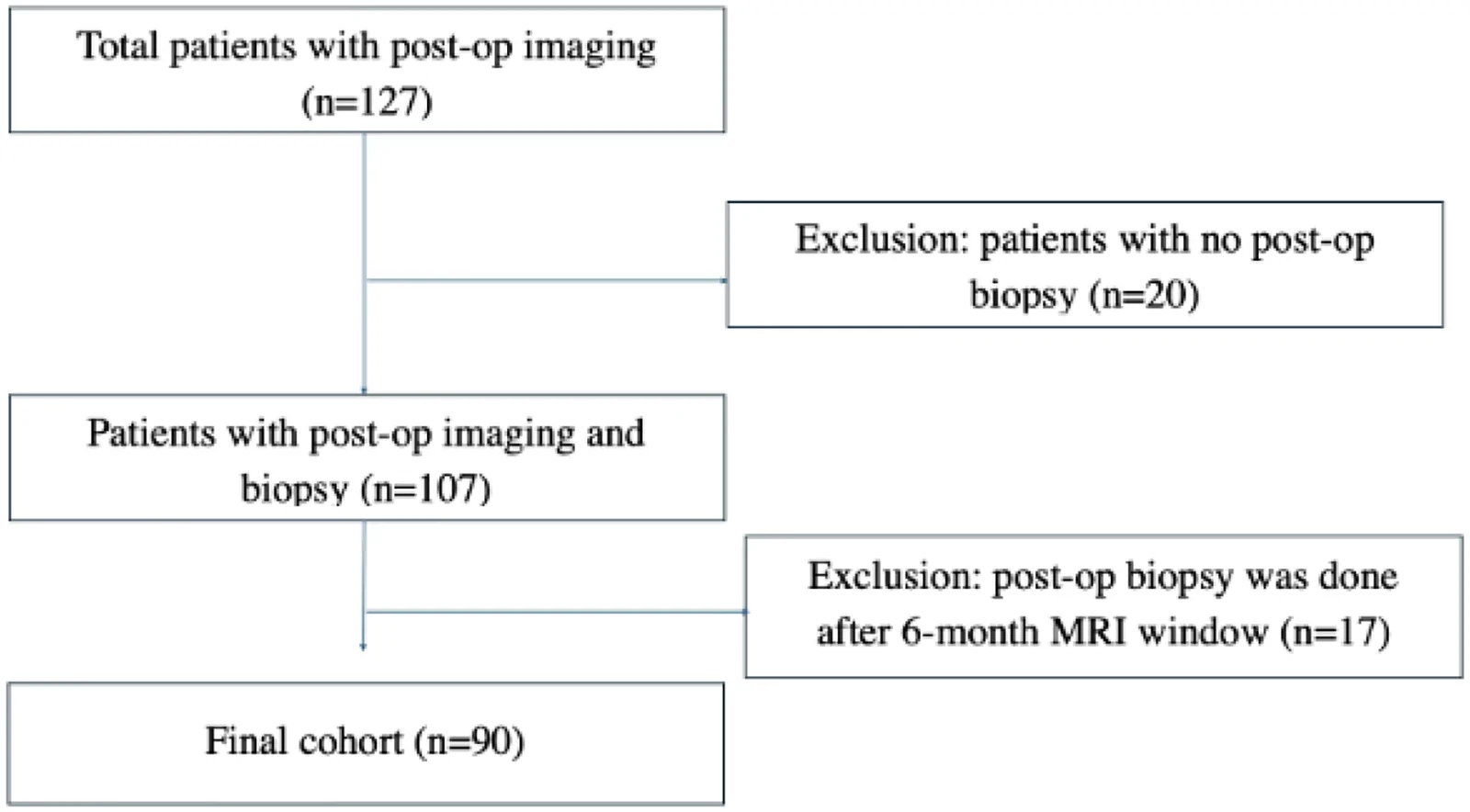
Patient inclusion criteria

**Figure 2:**
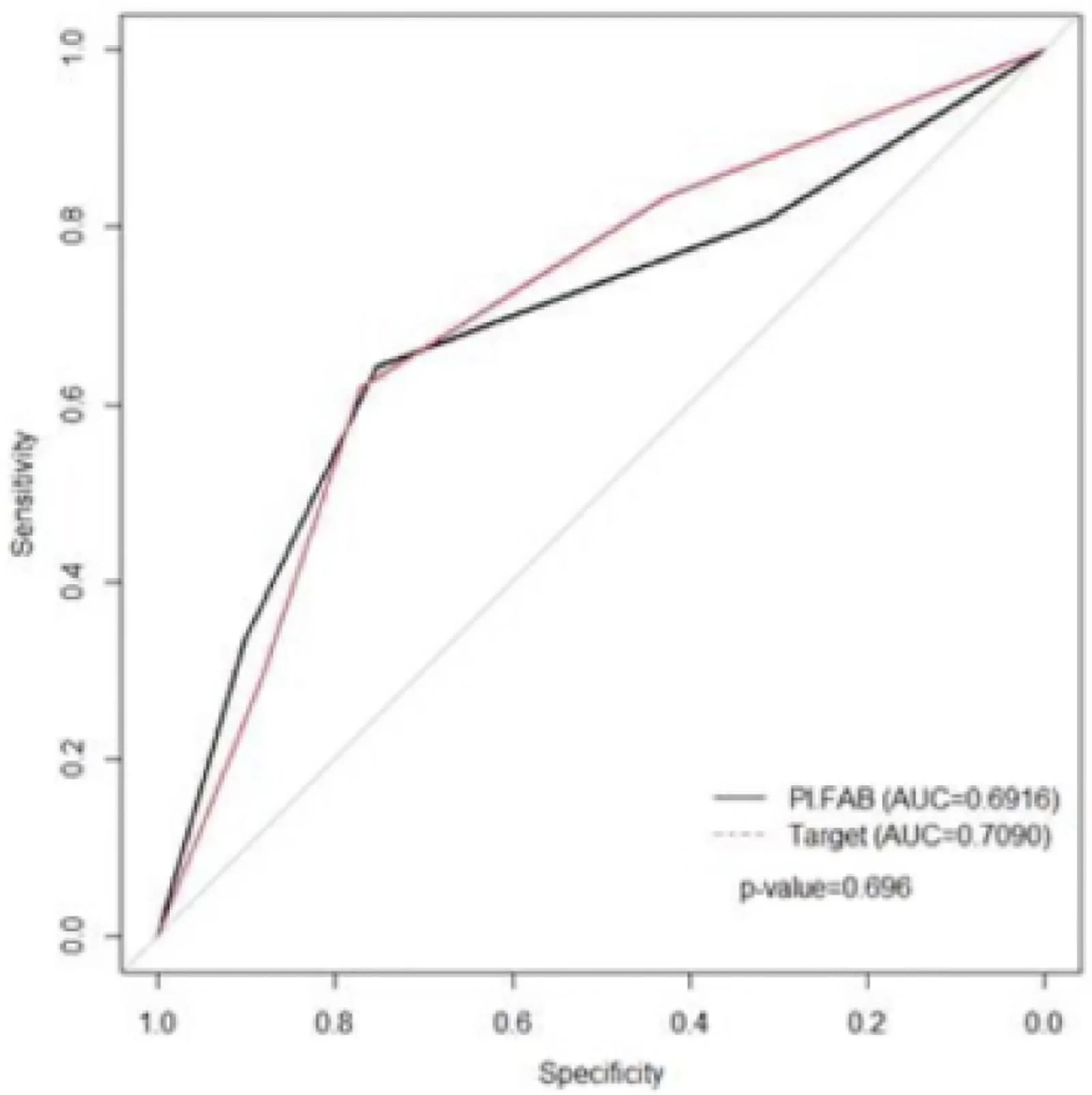
Diagnostic Performance by Scoring System Abbreviations: AUC, Area Under the Curve; PI-FAB, Prostate Imaging after Focal ABlation; TARGET, TransAtlantic Recommendations for prostate Gland Evaluation with magnetic resonance imaging after focal Therapy. Note: AUC for PI-FAB was 0.6916 and AUC for TARGET was 0.7090 when using a model that includes radiologist as a covariate. There was no statistically significant difference between AUC curves (p=0.696).

**Figure 3:**
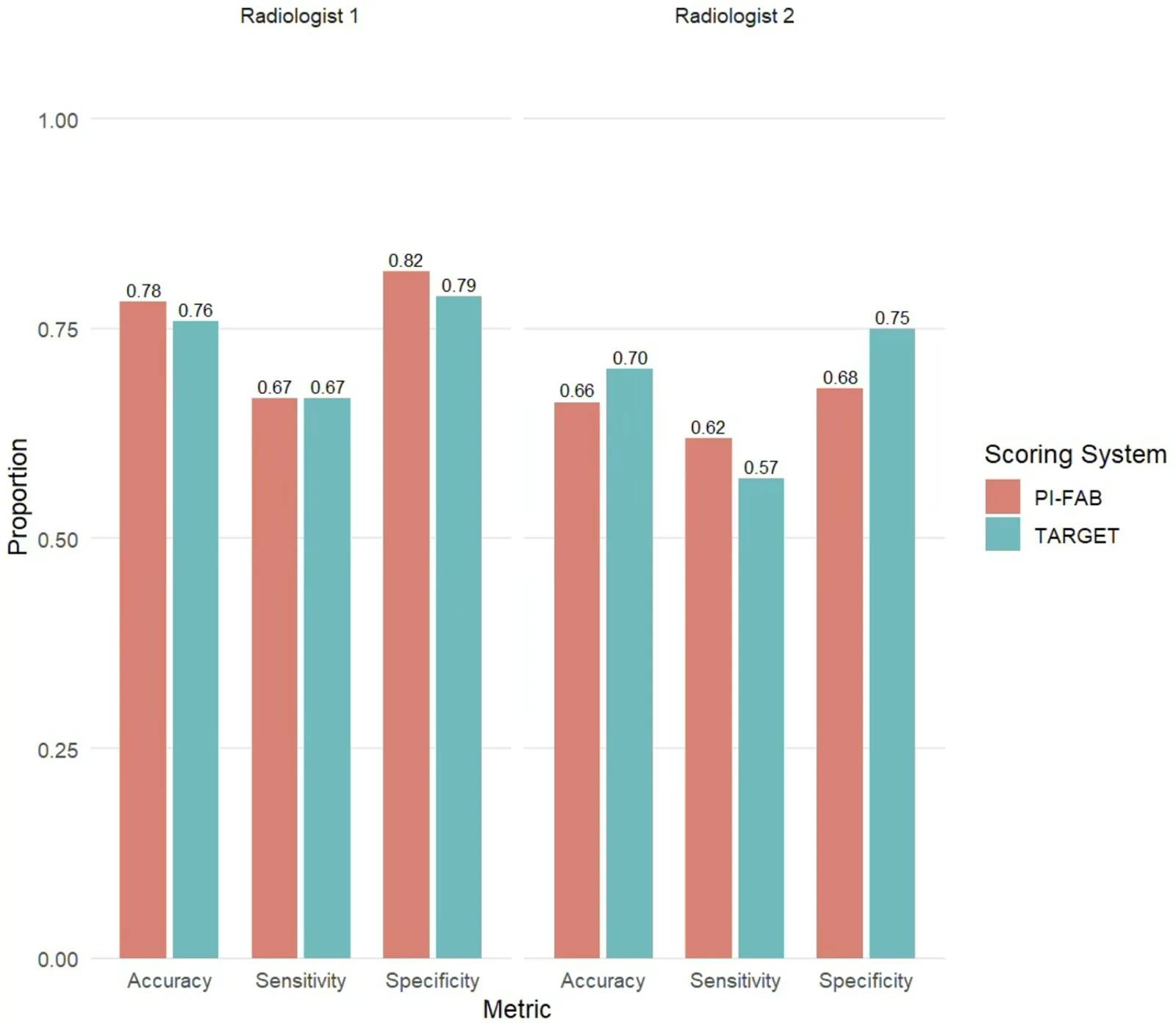
Diagnostic Performance by Reader and Scoring System

**Figure 4:**
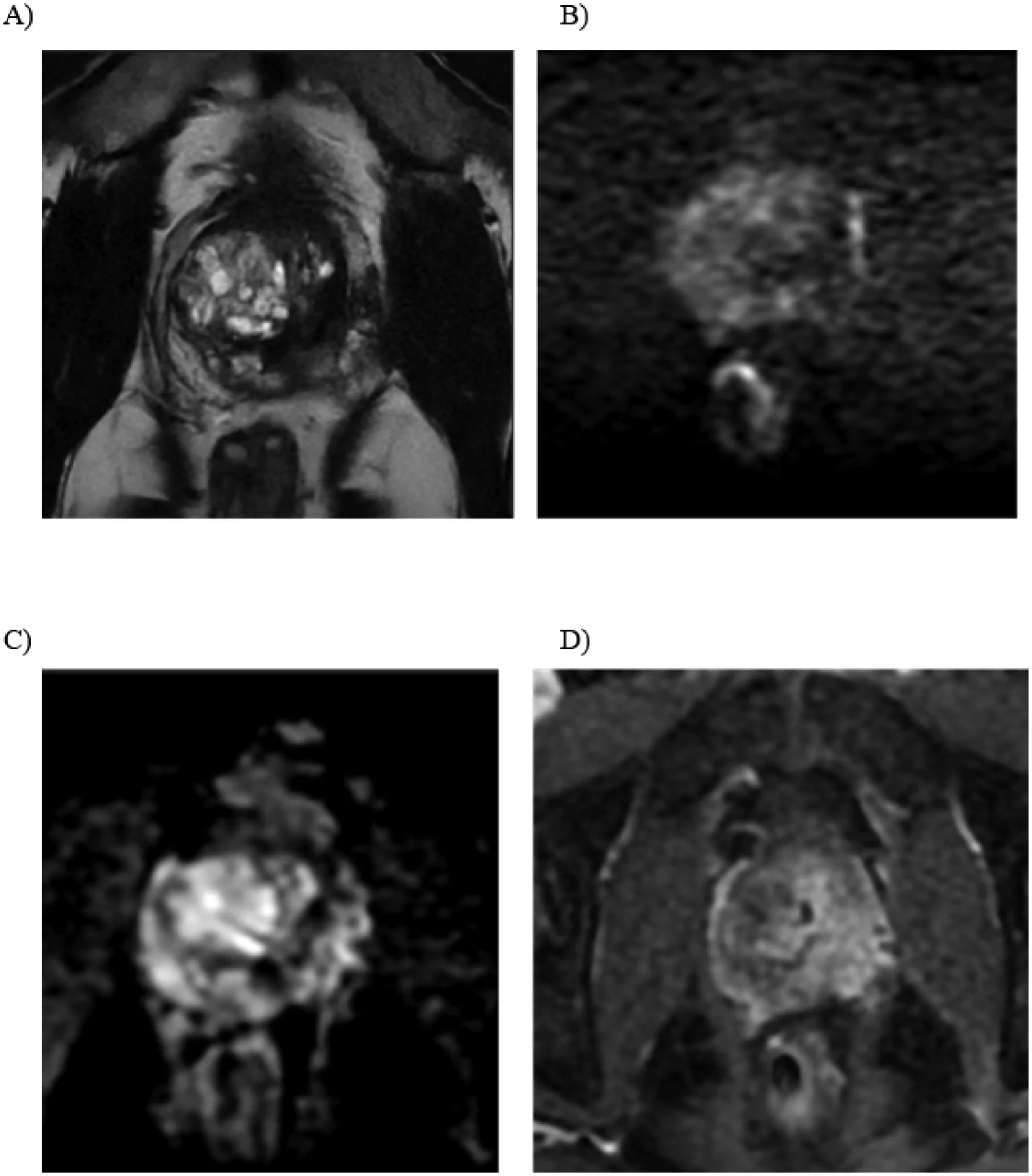
mpMRI imaging sequences post-focal therapy (A) T2, (B) DWI, (C) ADC, (D) DCE-MRI. MRI imaging sequences for a cancerous lesion on the left peripheral transition zone assigned a PI-FAB score of 3 and TARGET score of 5. DCE-MRI sequence was scored as 4 or 5, however linear diffusion restriction and dark T2 upgrades this lesion to a score of 5.

**Table 1:** Patient Characteristics (N = 90)

Age at FT, Median (IQR)	69 (64–72)
Race, N (%)	
Caucasian	73 (81.1)
African American	9 (10)
Other	8 (8.9)
Family history of PCa, N (%)	22 (24.4)
Prior PCa treatment, N (%)	11 (12.2)
PSA, ng/mL, Median (IQR)	6.0 (4.7–8.1)
Prostate volume, mL, Median (IQR)	40.5 (30.2–57.4)
No. MRI lesions, Median (IQR)	1 (1–2)
MRI primary lesion size, Median (IQR)	11 (8–15)
PI-RADS primary lesion, Median (IQR)	4 (4–5)
No. cores positive systematic biopsy, Median (IQR)	3 (1.5–4.5)
Biopsy Grade Group, N (%)	
1–2	61 (67.8)
3–5	29 (32.2)
Focal Therapy Technique, N (%)	
HIFU	57 (63.3)
Cryoablation	12 (13.3)
Water Vapor Ablation	12 (13.3)
IRE	8 (8.9)
TULSA-PRO	1 (1.1)
Clinical T stage, Median (IQR)	2 (2–2)
Post-FT csPCa, N (%)	22 (24)

**Table 2:** Diagnostic Performance Results

	PI-FAB						TARGET					
	Rad1			Rad2			Rad1			Rad2		
	Formula (n/N)	Value	95% CI	Formula (n/N)	Value	95% CI	Formula (n/N)	Value	95% CI	Formula (n/N)	Value	95% CI
**Sensitivity**	14/21	0.67	0.43–0.85	13/21	0.62	0.38–0.82	14/21	0.67	0.43–0.85	12/21	0.57	0.34–0.78
**Specificity**	54/66	0.82	0.70–0.90	38/56	0.68	0.54–0.80	52/66	0.79	0.67–0.88	42/56	0.75	0.62–0.86
**PPV**	14/26	0.54	0.33–0.73	13/31	0.42	0.25–0.61	14/28	0.5	0.31–0.69	12/26	0.46	0.27–0.67
**NPV**	54/61	0.89	0.78–0.95	38/46	0.83	0.69–0.92	52/59	0.88	0.77–0.95	42/51	0.82	0.69–0.92
**Accuracy**	68/87	0.78	0.68–0.86	51/77	0.66	0.55–0.77	66/87	0.76	0.65–0.84	54/77	0.7	0.59–0.80
**AUC**	-	0.74	0.63–0.86	-	0.65	0.53–0.77	-	0.73	0.61–0.84	-	0.66	0.54–0.78
**p-value**	< .0001	-	-	0.0348	-	-	< .0001	-	-	0.0171	-	-

Abbreviations: AUC, Area Under the Curve; PPV, Positive Predictive Value, NPV, Negative Predictive Value

## Data Availability

The data that support the findings of this study are available within the manuscript. Original data not openly available to protect patient privacy but available from the corresponding author upon reasonable request. Data are located in controlled access data storage at the University of Chicago.

## Abbreviations

FT: focal therapy
IQR: interquartile range
PCa: prostate cancer
PSA: prostate specific antigen
PIRADS: prostate imaging reporting and data system. Note: Values are presented as N (%) or median (IQR).
